# Role of BK human polyomavirus in cancer

**DOI:** 10.1186/s13027-018-0182-9

**Published:** 2018-04-05

**Authors:** Jorge Levican, Mónica Acevedo, Oscar León, Aldo Gaggero, Francisco Aguayo

**Affiliations:** 1 Programa de Virología, Instituto de Ciencias Biomedicas, Facultad de Medicina, Universidad de Chile, Santiago, Chile; 2 Departamento de Oncología Básico clínica, Facultad de Medicina, Universidad de Chile, Santiago, Chile; 3 Advanced Center for Chronic Diseases (ACCDiS), Universidad de Chile, Santiago, Chile

**Keywords:** Polyomavirus, Cancer, Oncoprotein

## Abstract

Human polyomaviruses (HPyV), which are small DNA viruses classified into the polyomaviridae family, are widely distributed in human populations. Thirteen distinct HPyVs have been described to date. Some of these viruses have been found in human tumors, suggesting an etiological relationship with cancer. In particular, convincing evidence of an oncogenic role has emerged for a specific HPyV, the Merkel cell polyomavirus (MCPyV). This HPyV has been linked to rare skin cancer, Merkel cell carcinoma (MCC). This finding may be just the tip of the iceberg, as HPyV infections are ubiquitous in humans. Many authors have conjectured that additional associations between HPyV infections and neoplastic diseases will likely be discovered. In 2012, the International Agency for Research on Cancer (IARC) evaluated the carcinogenicity of the BK virus (BKPyV), reporting that BKPyV is “possibly carcinogenic to humans.” This review explores the BKPyV infection from a historical point of view, including biological aspects related to viral entry, tropism, epidemiology and mechanisms potentially involved in BKPyV-mediated human carcinogenesis. In order to clarify the role of this virus in human cancer, more epidemiological and basic research is strongly warranted.

## Background

**Fig. 1:**
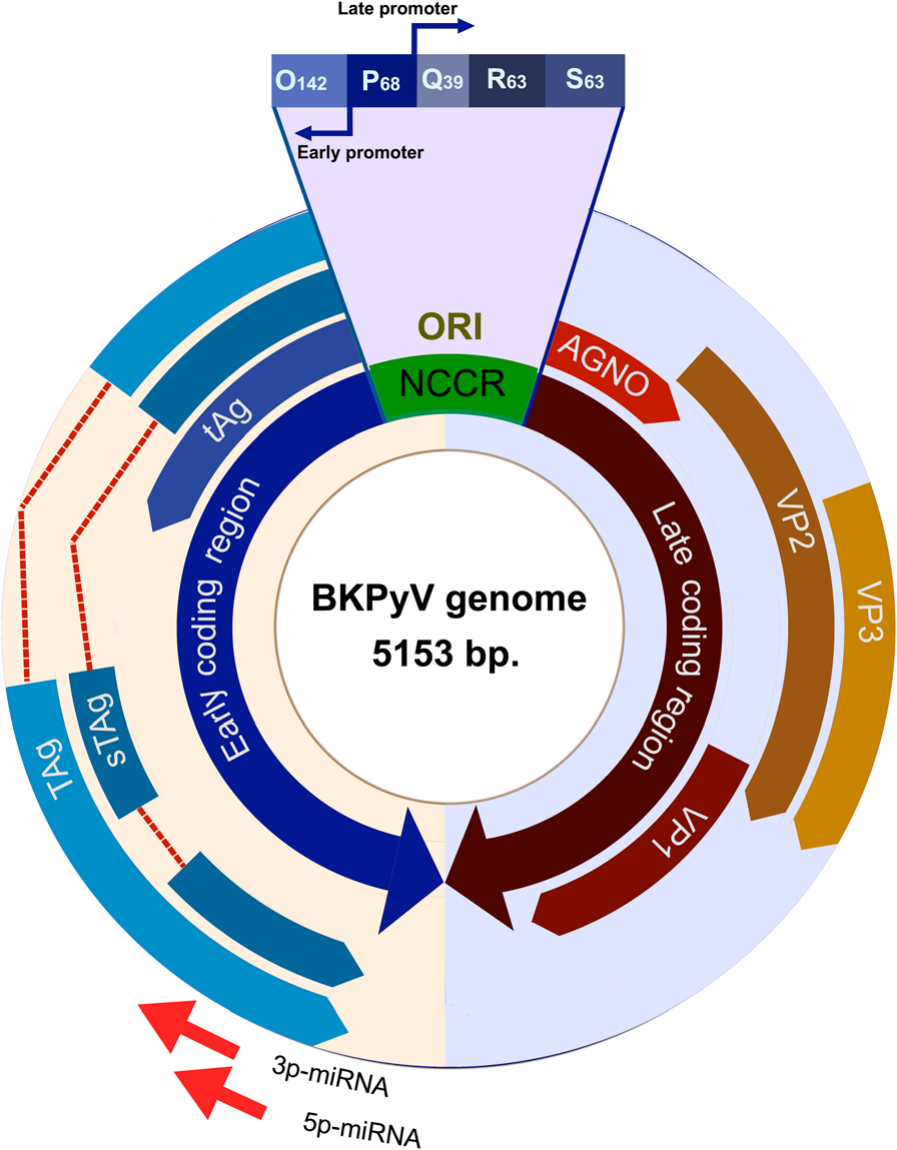
Genome map of BKPyV

Human polyomaviruses (HPyVs) are small, non-enveloped, double-stranded DNA viruses with approximately 5000-bp genome and icosahedral symmetry. These viruses belong to the polyomaviridae family. The HPyV genome encodes early small-t/large-T antigens as well as late structural proteins called VP1, VP2, VP3, and agnoprotein. The early region, which is transcribed before DNA replication begins, is composed of large T and small t antigen genes and the splice variants *T* = 135, *T* = 136, and *T* = 165 [1]. The late region is transcribed concomitant with DNA replication. The HPyV capsid harbors 72 pentamers of VP1, which interacts with the VP2/VP3 molecules associated with each pentamer [2]. In addition, these viruses encode a pre-miRNA for generation of two mature miRNAs [3, 4]. A non-coding control region (NCCR) is located between the oppositely-oriented transcriptional units that encode for early and late transcripts. The NCCR contains the promoters and enhancers for regulation of gene expression and harbors the replication origin (Ori) [5]. In BKPyV, JCPyV, and SV40, the agnoprotein is expressed from the 5’region of VP2 open reading frame. It is believed that this protein is involved in various functions related to the HPyV life cycle, such as regulating viral gene expression or inducing viral maturation [6, 7]. A scheme of the BKPyV structure is shown in Fig. 1. The functions of encoded viral products are summarized in Table 1.

**Table 1:** Function of BKPyV gene products

	BKPyV expression products	Function
Early	Large Tumour Antigen (Tag)	Cell cycle progression, inhibition of apoptosis, viral replication
	truncated Large T antigen (truncTAg)	Cell cycle progression, viral replication
	Minor T Antigen (tAg)	Cell cycle progression
	3p-miRNA	viral persistence
	5p-miRNA	viral persistence
Late	VP1	capsid structure (external), viral attachment and entry
	VP2	capsid structure (internal), involved in viral infectivity
	VP3	capsid structure (internal), involved in viral infectivity
	Agno protein	Life cycle (assembly, maturation, release)

In natural hosts, HPyVs establish a productive infection, while in heterologous, non-permissive hosts, the virus establishes latency with potential integration into the host genome (reviewed in [8]). HPyV infection typically occurs early in life, often through fecal-oral transmission, and persists throughout the lifespan [9]. With the development of new high-throughput sequencing techniques, fourteen HPyVs have been described, most of which were discovered in the last few years [10]. As HPyVs are ubiquitous, associations between these viruses and various pathologies are a focus of intensive research, especially the possible contributions of HPyVs to cancer etiology. Four polyomaviruses have been found to show oncogenic potential — SV40, BKPyV, JCPyV, and MCPyV — although there is strong evidence of such a link only in the case of MCPyV. This virus appears to play a role in a rare skin cancer, Merkel cell carcinoma [11]. A carcinogenic role has also been suspected for SV40, but the association remains controversial as no robust evidence has emerged. This virus was initially detected as a contaminant in the poliovirus vaccine, with many infections occurring between 1955 and 1963 [12]. This review evaluates the molecular mechanisms of BKPyV infection and its potential association with cancer.

### BK virus

#### Viral entry

During BKPyV infection, VP1 interacts with the α2, 8-SA-containing b-series gangliosides (GD1b/GT1b) for cell attachment [13]. A crystal-like complex of VP1 and the ganglioside GD3 is formed, with several points of contact between VP1 and two sialic molecules of a disialic acid ganglioside [14]. This model was tested using site-directed mutagenesis. It was concluded that a specific contact between the terminal sialic acid residue of GD3 and VP1 is essential for virus infection. Previous experiments carried out on African green monkey kidney cells suggest that caveolin is involved in BKPyV entry. However, the entry mechanism of BKPyV was recently re-examined in a primary culture of human renal proximal tubule epithelial cells. Using a siRNA strategy, it was demonstrated that BKPyV entry is caveolin- and clathrin-independent [15]. These findings, along with the fact that virus entry does not require actin polymerization, exclude other known alternative endocytic pathways and suggests that BKPyV utilizes an as-yet-uncharacterized endocytic pathway [15, 16]. After entering the cell, the virus must reach the nucleus for replication. This process depends on acidification and maturation of the endosome and involves retrograde transit of endocytic vesicles to the endoplasmic reticulum (ER) [17, 18]. Once in the ER, partial disassembly occurs. Viral particles escape to the cytoplasm, hijacking ER Derlin family proteins [18]. Finally, the virus enters the nucleus via the importin-α/β pathway, guided by nuclear localization signals present in the minor capsid proteins VP2 and VP3 [19].

#### Tropism and epidemiology

BKPyV infection is a widely-distributed strict anthroponosis. The primary infection most often occurs in early childhood, with a seroprevalence of 65–90% in 5–9 year-old children [20]. Following primary infection, BKPyV persists in the kidneys. If the host becomes immunosuppressed, the virus causes significant morbidity. For instance, BKPyV causes hemorrhagic cystitis and nephropathy (BKAN) in bone marrow and renal transplant patients, respectively [21, 22]. BKPyV genomes have also been detected in a wide spectrum of normal tissues including liver, stomach, lungs, parathyroid glands, lymph nodes, brain, peripheral blood mononuclear cells, bladder, uterine cervix, vulva, prostate, lips and tongue [23]. Although the transmission mechanism is not completely elucidated, the high resistance of BKPyV to environmental inactivation and its presence at high concentrations in human sewage and other water sources suggest fecal-oral transmission [24]. In this respect, it was reported that salivary glands and oropharyngeal cells are not involved in BKPyV persistence, suggesting that digestive tract would be important for viral transmission [25]. In addition, Taguchi et al. showed a vertical transmission of this virus only in the case of primary BKV infection of serologically negative pregnant women [26]. Moreover, it was reported the presence of BKPyV in 7 out 10 specimens of aborted fetuses suggesting the possibility of transplacental transmission [27]. Once the virus enters to the body, probably peripheral blood leukocytes (PBLs) transport BKPyV to different sites and organs [28].

Upon BKPyV infection in a permissive host cell, early gene expression leads to DNA replication, followed by late gene expression, production of progeny viral particles and cell death. Permissive cells for viral replication are kidney cells such as Vero (African green monkey kidney) [29], HEK293 (human embryonic kidney cells) [30] or RPTE (primary human renal proximal tubule epithelial cells) [31]. In addition, it has been reported that some salivary glands cells are permissive for BKPyV infection [32].

In a non-permissive cell, BKPyV lytic replication is blocked, and abortive infection may result in oncogenic transformation [33].

Although the oncogenic activity of BKPyV is well-documented in laboratory settings, there is no conclusive evidence of a causal relationship between BKPyV and cancer in human beings. This relationship is difficult to demonstrate in ecological contexts for several reasons: first, the viral agent has a high prevalence in the general population; second, there are a wide range of human tissues in which the virus can be detected; and third, the virus has the ability to remain in a latent state for long periods, with occasional reactivations.

#### Role in carcinogenesis

The oncogenic properties of BKPyV are well-demonstrated in in vitro and in vivo experimental models. The transforming activity has been mapped in the early region of the BKPyV genome, which encodes two viral oncoproteins: the large T-antigen (TAg) and the small t-antigen (tAg). These viral products induce alterations in the normal cell cycle, ultimately leading to cell immortalization and neoplastic transformation [34]. In one study, transfection of embryonic fibroblasts or cells cultured from kidney or brain tissues of diverse mammalian origin with complete or sub-genomic fragments of BKPyV DNA, containing the early coding region, lead to cell transformation [23]. In addition, transformation with a recombinant construct containing the BKPyV TAg gene and the activated c-Ha-ras oncogene-induced neoplastic transformation at early passages in hamster embryo cells with higher efficiency when compared to independently-transfected genes, suggesting a cooperative effect of the two oncogenes in early carcinogenesis [23]. Moreover, continuous expression of functional TAg is required for the maintenance of BKPyV transformation in hamster and mouse cells [23].

BKPyV large TAg is a highly multifunctional protein that can bind various cellular proteins, altering signaling pathways involved in cell cycle control. The most frequently-studied cellular targets of TAg are the p53 family proteins and pRb tumor suppressor proteins. The interaction between BKPyV TAg and p53 results in the inactivation of this protein, interfering with the response to DNA damage and inducing the unscheduled onset of the S-phase [35]. Therefore, BKPyV TAg drives the cell to override a key cell cycle checkpoint, favoring the accumulation of genetic alterations during each cell replication cycle [23, 36, 37]. In addition, the interaction between BKPyV TAg and pRb leads to the release and nuclear translocation of the E2 factor (E2F) family of transcription factors and subsequent expression of genes, inducing quiescent cells to enter the S-phase [37, 38]. The other early gene product of BKPyV, the small tAg, plays an important role in transformation by inhibiting protein phosphatase 2A (PP2A), an essential tumor suppressor in numerous death-signaling pathways. The tAg protein shows two conserved cysteine cluster motifs (CXCXXC) that are thought to be involved in the interaction with PP2A [39]. Indeed, the catalytic (36-kDa) and regulatory (63-kDa) subunits of PP2A have been co-immunoprecipitated with anti-tAg from BKPyV-infected human embryonic kidney (HEK) cells [40]. By inactivating this negative regulator, BKPyV tAg can activate signaling pathways that promote cell proliferation, such as mitogen-activated protein kinase (MAPK) [23, 33, 39].

Early reports demonstrated that BKPyV is highly oncogenic in rodents [41, 42]. Assays conducted in newborn hamsters, mice, and rats inoculated with the virus showed that these animals developed tumors at various locations that contained BKPyV DNA sequences, either integrated into the host genome or in a free episomal form with constitutive TAg expression [23]. In addition, animals injected with BKPyV frequently developed ependymomas, pancreatic islet tumors, osteosarcomas, fibrosarcomas, liposarcomas, osteosarcomas, nephroblastomas and gliomas [23]. Transgenic mice expressing the BKPyV early genome region were found to develop highly tissue-specific tumors. Small et al. showed that these animals developed primary hepatocellular carcinomas and renal tumors [43]. Dalrymple and Beemon also observed two types of alterations: enlarged thymuses and renal adenocarcinomas. Moreover, BKPyV TAg expression in these mice was restricted to the epithelial cells of the kidney tumors and enlarged thymuses [44].

Although the transforming ability of BKPyV is well-documented in experimental rodent models, definitive transforming activity is not always observed in human and primates. The transformation of HEK cells by BKPyV is not efficient and is often abortive, and features of the transformed phenotype are not fully displayed [37, 45]. For instance, BKPyV TAg was able to induce serum-independent growth in BSC-1 African green monkey kidney cells but was unable to induce anchorage-independent growth in soft agar [37]. In addition, BKPyV TAg activity is lower in BKPyV-infected BSC cells than the TAg activity expressed by the SV40 virus under the same conditions. TAg expressed by BKPyV is not sufficient to completely capture the Rb family of proteins. It has been proposed that the difference between SV40 and BKPyV TAg activity may be due to lower expression levels of the BKPyV promoter and enhancer elements, which share only 40% homology with the SV40 promoter region. Alternatively, this finding may be a consequence the greater instability of the BKPyV TAg as compared to SV40 TAg protein [37]. These findings suggest that while BKPyV TAg may modulate cellular growth through direct interactions with critical regulatory proteins, additional events are required for complete transformation. These events could be mutations or alterations of the viral promoter-enhancer elements, leading to increased expression of early genes and a consequent increase in transforming activity [46]. In support of this model, integration of early-region viral sequences into the host genome has been shown to account for the difference between serum-independent growth and full transformation in BKPyV-infected human embryonic kidney cells [47]. This integration event could result in positioning of BKPyV TAg coding sequence under the control of nearby cellular promoter-enhancer elements.

An alternative model for the role of BKPyV TAg in oncogenesis involves the first step in which BKPyV TAg binds to or inactivates tumor suppressor proteins, with a second step leading to cellular oncogene activation. This model is supported by studies showing that human embryonic kidney cells persistently infected with BKPyV exhibited a semi-transformed phenotype and that full transformation resulted from the additional presence of activated Ha-ras oncogenes [48]. Therefore, p53 inactivation by BK TAg may lead to random mutational events that could activate cellular oncogenes or inactivate other tumor suppressor genes. Moreover, it has been shown that BKPyV TAg induces chromosomal instability in human embryonic fibroblasts, characterized by gaps, breaks, dicentric and ring chromosomes, deletions, duplications and translocations [49]. Consistent with early participation of BKPyV TAg in tumorigenesis, there is evidence that these alterations occur before immortalization [23]. Once chromosomal alterations are fixed into the host cells, viral sequences may be dispensable for the maintenance of transformation and may be lost in the neoplastic tissues.

Various authors have detected BKPyV genetic material in a wide range of human tumors [23, 33]. For instance, the early BKPyV genome region has been detected in brain tumors, osteosarcomas, Ewing’s tumors, neuroblastomas and genitourinary tract tissues tumors, including prostatic and bladder cancer [23, 33, 50, 51]. In contrast, other authors reported no association between BKPyV DNA and tumors [33, 52–56]. In any case, the mere presence of BKPyV DNA does not necessarily reflect a neoplastic involvement of the virus. In some cases, BKPyV may not be directly involved in the development of cancer, but instead, play a role as a co-factor in the carcinogenic process. For instance, the virus may co-infect cells that were previously infected by another oncogenic virus, increasing susceptibility to cancer. In fact, HPyVs have been detected in various tissues that are susceptible to transformation by HTLV-I, HCV, HPV, EBV, HHV-8 and HBV [57]. In addition, recent reports have documented the presence of BKPyV DNA in association with HPV16 in high-grade cervical squamous intraepithelial lesions [58]. The association of BKPyV with precancerous cervical lesions suggests that this virus could be involved in HPV16-induced cell transformation. Alternatively, BKPyV might benefit from proliferative enhancement of HPV16-positive cells in precancerous cervical cells. Further experimental studies and clinical observations are needed to verify whether this putative transformation mechanism involving BKPyV and HPV occurs in cervical cancer [58].

Early findings established that BKPyV has a tropism for certain cell types and that this agent can establish a persistent or latent infection in the kidney and urinary tract [59]. Therefore, carcinomas that affect this anatomical zone are likely candidates for associations with BKPyV. Among these diseases, renal cancer, urothelial bladder cancer, and prostatic cancer have been extensively studied [23, 60].

The contribution of BKPyV to the etiology of bladder carcinoma in immunocompetent individuals is not well-established. Some studies demonstrate BKPyV DNA sequences at high frequencies in bladder carcinoma [61, 62]. However, these studies were small case series that either lacked a control group or relied entirely on antibody seroprevalence [63]. In a multi-center study, Polesel et al. found similar a prevalence of the viral DNA in a group of 114 transitional bladder carcinoma cases and a group of 140 hospital controls. This result does not support the role of BKPyV in bladder cancer among immunocompetent individuals [64].

On the other hand, there are reports that link BKPyV with metastatic bladder carcinoma in immunosuppressed transplant recipients [65–67]. In a retrospective study, Roberts et al. reported that while no positive BKPyV TAg urothelial carcinomas were found in a series of non-transplanted patients (0/20), strong nuclear staining for TAg was seen in the urothelial carcinoma of one renal transplant patient [68]. This data indicates that although associations between BKPyV and these tumors are rare, the virus may have a tumorigenic role in some cases. In addition, in a retrospective review of kidney transplant patients, Chen et al. reported that 6/864 patients developed polyomavirus-associated nephropathy (PVAN). Malignancy occurred in 5/6 PVAN patients, suggesting that patients who develop PVAN are at significantly higher risk of developing cancers, including transitional cell bladder carcinoma [66, 69]. Although urothelial carcinomas expressing BK TAg are quite rare [68], these cancers show some distinct features. These tumors are high-grade and invasive. Lesions can arise in the renal allograft or the host urothelial tissue. While TAg is strongly expressed in the tumor cells, the late structural proteins are not expressed, and no viral replication is observed. This finding suggests that the possible oncogenic mechanism involves deregulation of the proliferation inducer TAg [70]. Recent findings using deep sequencing analysis from a high-grade BKPyV-associated tumor expressing TAg have revealed viral DNA integrated into the host genome [71, 72]. While the insertion site seems to be nonspecific, the virus genome linearization break-point was situated in the late gene coding region. This interruption accounts for the blockage of viral replication and suggests a concomitant disruption of regulatory feedback signals that control TAg expression [71, 72]. Moreover, Seo et al., (2008) reported that BKPyV codes a pre-miRNA hairpin at the 3′ end of the late region [73]. The maturation of this element gives rise to two miRNAs, 5p-miRNA and 3p-miRNA, which are perfectly complementary to Tag-coding mRNA. Whether these miRNAs are functional during BKPyV infection and how this regulatory disruption contributes to BKPyV-mediated cell transformation is currently under intense study.

Prostate cancer (PCa) is one of the leading causes of cancer deaths in men worldwide, and its relationship with BKPyV infection has been studied by several groups in recent years [74–80]. Monini et al. detected BKPyV in approximately 60% of cancerous and healthy prostates, and the viral load was found to be significantly higher in neoplastic as compared to non-neoplastic tissue [74]. Das and Russo found similar detection rates in PCa cases, with a significantly lower prevalence in controls [76, 77, 81]. On the other hand, other authors disagree with these results. Lau et al., using in situ, detected BKPyV in only 2/30 prostatic adenocarcinomas, and no TAg expression was detected in neoplastic tissue [79]. Similarly, Sfanos et al. of 338 analyzed total samples from 200 patients for BKPyV DNA and detected only one positive sample [82]. In Chile, our group found only 6/69 (8.7%) BKPyV DNA-positive prostate carcinomas, and the TAg transcripts were detected in 2/6 (33%) of BKPyV positive cases [83]. These apparently contradictory data can be partially explained due to the variable sensitivity and specificity of the detection methods used in each study. However, this finding may also suggest that the virus is dispensable at late stages of the disease, and it may be cleared from the lesion. In this context, it has been postulated that BKPyV constitutes an important factor for early prostate tumorigenesis [77]. Although p53 and pRb proteins are implicated in PCa, there is a low incidence of mutations in these genes during early stages of the disease [84]. This finding has led to the suggestion that a human oncogenic virus such as BKPyV may be implicated in the inactivation of these tumor suppressor proteins at early stages of tumorigenesis [76, 77]. There is growing evidence supporting this model. Using a combination of Laser Capture Microdissection (LCM) and molecular biology techniques, BKPyV DNA has been detected in the epithelial cells of benign and proliferative inflammatory atrophy (PIA) and prostate intraepithelial neoplasia (PIN), entities that have been postulated to be the early transition step toward overt PCa [76, 77, 85, 86]. In addition, using double immunofluorescence labeling with anti-p53 and TAg antibodies in BKPyV positive prostate tumor tissue sections, it has been observed that the two proteins colocalize in the cytoplasm. While the typical localization of both proteins is nuclear, the cytoplasmic localization suggests a functional inactivation mechanism by sequestration [81]. Moreover, p53 genes from atrophic cells expressing TAg are frequently wild-type, whereas tumor cells expressing detectable nuclear p53 contain a mix of wild-type and mutant p53 genes. This finding suggests a possible tumorigenic mechanism in which the TAg inactivates p53 in the atrophic cells, increasing susceptibility to genetic alterations, including tumor suppressor gene mutations that may result in early prostate cancer progression. This model is consistent with a “hit-and-run” carcinogenesis mechanism [87]. After cell transformation, the loss of BKPyV in the tumor cells could be due to selection against TAg by the immune system, dilution of viral episomes due to lack of replication or pro-apoptotic effects mediated by TAg that are not compatible with the other growth control mutations in the tumor cells, resulting in selection against TAg expression [23, 33, 76, 77, 81]. Nonetheless, the “hit-and-run” mechanism is difficult to defend experimentally. Taken together, a carcinogenic role of this virus has been difficult to demonstrate. Some arguments for a carcinogenic and for a non-carcinogenic role of BKPyV in human cancer are summarized in Table 2.

**Table 2:** Evidences for carcinogenic and non-carcinogenic role of BKPyV

Evidences of BKPyV carcinogenicity	Evidences for a non-carcinogenic role
Viral oncogenes are expressed in tumors	Poor and not efficient transforming activity in human cells
Tumors developed in in vivo models	Ubiquitous distribution in normal human cells and tissues
Transforming properties in in vitro models	Variable BKPyV presence in tumors among different studies
BKPyV alterations occur before immortalization	
BKPyV genome detected in human tumors	

## Conclusion

The challenge now is to devise investigative strategies that might lead to conclusive evidence that would allow us to confirm or exclude the role of BKPyV in the development of tumors. Thus, more epidemiological and experimental studies are strongly required. In addition, the possibility of interaction with other host-related factors, infectious agents or environmental components for carcinogenesis warrants more investigation.

## Abbreviations

BKPyV: BK polyomavirus
HPyV: Human polyomavirus
PCa: Prostate cancer
SV40: Simian Virus 40
TAg: Major T antigen
tAg: Minor T antigen
